# Investigation of Temperature Sensitivity of a Polymer-Overlaid Microfiber Mach-Zehnder Interferometer

**DOI:** 10.3390/s17102403

**Published:** 2017-10-21

**Authors:** Young-Geun Han

**Affiliations:** Department of Physics and the Research Institute for Natural Sciences, Hanyang University, Seoul 133–791, Korea; yghan@hanyang.ac.kr; Tel.: +82-2-2220-4552

**Keywords:** microfibers, microfiber Mach-Zehnder interferometers, thermal expansion effect, thermo-optic effect, temperature sensors

## Abstract

The temperature sensitivity of the free spectral range (FSR) for a polymer-overlaid microfiber Mach-Zehnder interferometer (MZI) is investigated both theoretically and experimentally. The waist diameter of the optical microfiber can be controlled to alter the thermal expansion and optic properties of the polymer-coated MZI. Inserting an optical microfiber with a strong evanescent field into the MZI, a low index polymer with high thermal characteristics is deposited on the surface of the microfibers to realize a polymer-overlaid microfiber MZI. It was found that the thermal expansion factor in the proposed MZI plays an important role in the temperature sensitivity of the FSR. The temperature sensitivity of the polymer-overlaid microfiber MZI is improved, which is measured to be −8.29 nm/°C at 25 °C. The optical transmission spectrum of the polymer-overlaid microfiber MZI is converted to the spatial frequency spectrum via fast Fourier transform. The temperature sensitivity of the spatial frequency in the proposed polymer-overlaid MZI is estimated to be 18.31 pm^−1^ °C^−1^, which is 17 times higher than that of the microfiber MZI without polymer coating (1.04 pm^−1^ °C^−1^).

## 1. Introduction

Fiber-optic interferometric sensors have been widely investigated for their various applications to mechanical, chemical, and biological measurement [1]. Fiber-optic Mach-Zehnder interferometers (MZIs) in particular have demonstrated the large potential of various sensing probes because of their numerous advantages, such as electromagnetic immunity, light weight, ease of fabrication, and compactness [2,3,4,5,6,7,8]. Many techniques to enhance the performance of MZI-based sensors have been proposed, including a micro-cavity inside the fiber [9,10,11], large lateral offset splicing [12], and a special fiber [13]. Recently, optical microfibers with a strong evanescent field have been proposed and applied to various physical sensors such as current sensing probes [14], compact refractometers [15], bidirectional bending sensors [16], coil resonators [17], and magnetic field sensors incorporating a fiber loop mirror [18]. Compared with conventional MZI-based sensing probes, inserting an optical microfiber into the MZI can make the microfiber MZI sensitive to external perturbations [19,20,21,22,23,24]. The temperature sensitivity of the microfiber MZI, however, has not yet been sufficiently investigated. 

In this paper, a polymer-overlaid microfiber MZI is fabricated by coating the microfiber with low index polymer. By considering the waist diameter of the microfiber, the thermal expansion, and the thermo-optic factors in the polymer-overlaid MZI, the temperature sensitivity of the proposed microfiber MZI is investigated. To initially configure a microfiber MZI, a microfiber with a waist diameter of 10 μm is fabricated. Then, low index polymer with high thermal quantiles is deposited on the surface of the microfiber, and consequently the polymer-overlaid microfiber MZI is realized. By considering the contributions of the thermal properties of polymer-coated microfibers to the temperature sensitivity of the proposed microfiber MZI, it was determined that the thermal expansion factor is higher than the thermo-optic factor. Further, the temperature sensitivity of the free spectral range (FSR) in the polymer-overlaid microfiber MZI is improved, and is measured to be −8.29 nm/°C at 25 °C. The transmission spectra of the proposed polymer-overlaid microfiber MZI is transformed to the spatial frequency spectra using fast Fourier transform (FFT). Compared with the temperature sensitivity of the microfiber MZI without the low index polymer overlay (1.04 pm^−1^ °C ^−1^), the temperature sensitivity of the spatial frequency is effectively increased via the proposed polymer-overlaid microfiber MZI, and is measured to be 18.31 pm^−1^ °C ^−1^ in the temperature range of 25 to 80 °C.

## 2. Fabrication of the Polymer-Overlaid Microfiber MZI

**Figure 1:**
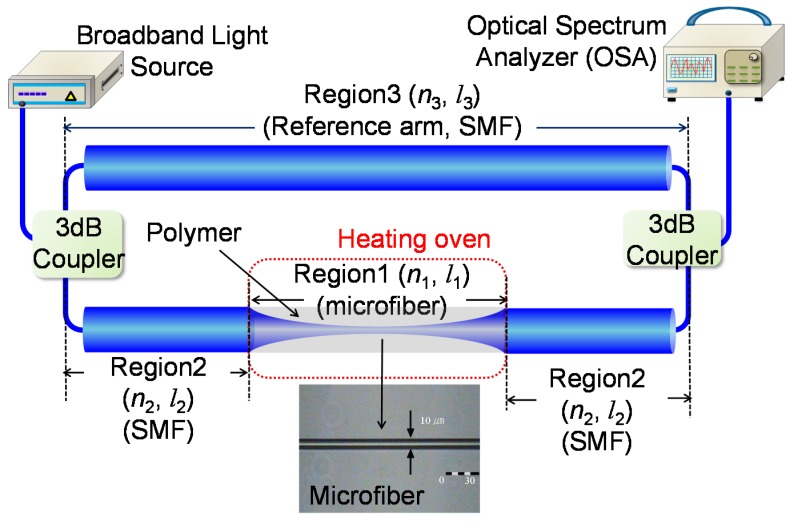
Experimental scheme for the polymer-overlaid microfiber MZI.

**Figure 2:**
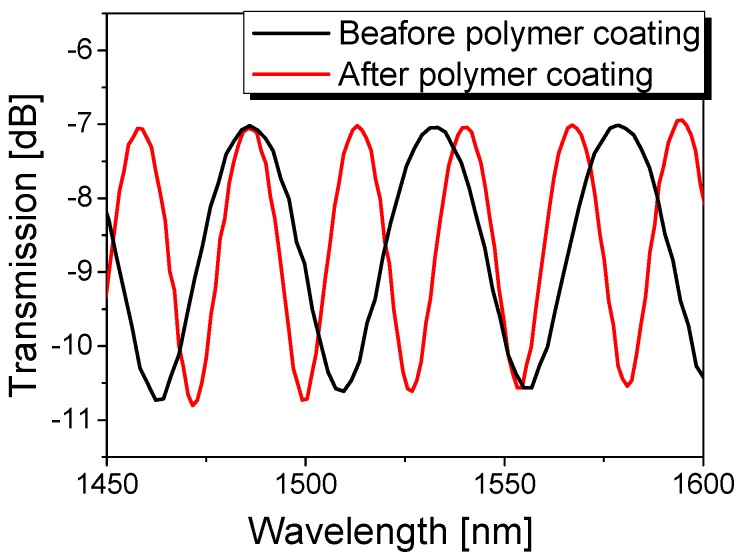
Interference spectrum of the polymer-overlaid microfiber MZI.

Figure 1 depicts the experimental configuration of the polymer-overlaid microfiber MZI, which includes two 3-dB couplers, as well as reference and sensing arms. The reference arm (Region 3) is composed of a conventional single-mode fiber (SMF) with a diameter of 125 μm and a length of 46.8 cm (*l*_3_). The sensing arm contains a polymer-overlaid microfiber (Region 1) between two SMFs (Region 2) with a total length of 40.6 cm (*l*_2_). A flame brushing technique was exploited to adiabatically taper an SMF to gradually reduce the waist diameter [25]. The SMF fixed on the motorized translation stage was elongated by exposure to a computer-controlled heater. High-order modes in the microfiber can be excited from the fundamental core mode by non-adiabatically tapering the SMF, resulting in an in-line modal interferometer depending on the waist diameter of the microfiber [25,26,27]. The waist diameter and the length (*l*_1_) of the fabricated microfiber were measured to be ~10 μm and ~6.3 cm, respectively. Then, the microfiber was spin-coated using a UV-curable polymer with a low refractive index (PC-373, SSCP, *n* = 1.375) and, consequently, the polymer-overlaid microfiber MZI was realized. Figure 2 shows the experimental results of the transmission spectra of the microfiber MZI (black line) and the polymer-overlaid microfiber MZI (red line). The free spectral range (FSR) of the microfiber MZI at room temperature (25 °C) changed from 47.5 to 27.8 nm before and after polymer coating, respectively. Since the polymer coating overlay increased the effective refractive index of the microfiber, the optical path length difference of the arms in the proposed microfiber MZI would also be increased. Consequently, the FSR should be diminished by coating the microfiber MZI with low index polymer. 

## 3. Theoretical Analysis of the Temperature Sensitivity of the FSR in the Polymer-Overlaid Microfiber MZI

In general, the FSR (Δ*λ*) of the MZI can be written as [7]:

(1) $$\Delta \lambda =\frac{\lambda_{1}\lambda_{2}}{\Delta \phi }$$

where *λ*_1_ and *λ*_2_ represent the two adjacent maximum and minimum wavelengths, respectively, and Δ*φ* is the optical path length difference between the two arms in the MZI. From Equation (1), the dependence of the FSR on the temperature (T) can be described by:

(2) $$\frac{\partial \left(\Delta \lambda \right)}{\partial T}=\frac{\partial }{\partial T}\left(\frac{\lambda_{1}\lambda_{2}}{\Delta \phi }\right)=-\frac{\lambda_{1}\lambda_{2}}{{\left(\Delta \phi \right)}^{2}}\left(\frac{\partial \left(\Delta \phi \right)}{\partial T}\right)$$

The proposed microfiber MZI has two different optical sections that consist of polymer-overlaid microfibers and SMFs. The optical path length difference (Δ*φ*) between the sensing and the reference arms in the polymer-overlaid MZI can be described as

(3) $$\Delta \phi =\phi_{1}+\phi_{2}-\phi_{3}=n_{1}l_{1}+n_{2}l_{2}-n_{3}l_{3}$$

where *φ*_1_, *φ*_2_, and *φ*_3_ are the optical path lengths in Regions 1, 2, and 3, respectively, and *n*_1_, *n*_2_, *n*_3_ and *l*_1_, *l*_2_, *l*_3_ are the effective refractive indices and the physical lengths of Regions 1, 2, 3, respectively. Since Region 1 (microfiber), when viewed as a sensing element, is only exposed to different temperatures via a heating oven, the optical quantities of Regions 2 and 3 (*n*_2_, *n*_3_, *l*_2_, and *l*_3_) cannot be changed by changing the temperature. The derivative of the optical path length difference with respect to temperature can be written as:

(4) $$\frac{\partial \left(\Delta \phi \right)}{\partial T}=n_{1}\frac{\partial l_{1}}{\partial T}+l_{1}\frac{\partial n_{1}}{\partial T}$$

By substituting Equation (4) into Equation (2), we obtain:

(5) $$\frac{\partial (\Delta \lambda )}{\partial T}=-\frac{\lambda_{1}\lambda_{2}}{{\left(\Delta \phi \right)}^{2}}\left(\underset{TE}{\underset{︸}{n_{1}\frac{\partial l_{1}}{\partial T}}}+\underset{TO}{\underset{︸}{n_{1}\frac{\partial n_{1}}{\partial T}}}\right)$$

In Equation (5), it is evident that two dominant factors, the thermal expansion (TE) and the thermo-optic factors (TO), play an important role in determining the temperature sensitivity of the FSR in the polymer-overlaid microfiber MZI. By definition of the thermal expansion coefficient (*α* = *∂l*/*l*∂*T*), the thermal expansion (TE) term in Equation (5) can be modified as:

(6) $$n_{1}\frac{\partial l_{1}}{\partial T}=\alpha_{1}l_{1}n_{1}\approx \alpha_{Coating}l_{1}n_{1}$$

where *α*_1_ is the thermal expansion coefficient of the polymer-coated microfiber (Region 1), which consists of two terms, *α*_Silica_ and *α*_Coating_. Microfibers with a small waist diameter of less than 10 μm can be largely considered as a fused silica waveguide [28,29]. To improve the thermal expansion factor in Equations (5) and (6), we exploited the low index coating polymer with a high thermal expansion coefficient (*α*_Coating_ = 400 × 10^−6^/°C), which is 800 times higher than that of fused silica (*α*_Silica_ = 0.55 × 10^−6^/°C). Consequently, we approximated *α*_1_ in Equation (6) and found it to be mostly determined by the thermal expansion coefficient of the low index polymer (*α*_Coating_); this is because the thermal expansion coefficient of silica (*α*_Silica_) is negligible. The thermo-optic factor (TO) in Equation (5) can be written as [7,28,29]:

(7) $$l_{1}\frac{\partial n_{1}}{\partial T}=l_{1}\left(\sigma_{\mathrm{Silica}}+\sigma_{\mathrm{Coating}}\frac{\partial n_{1}}{\partial n_{\mathrm{Coating}}}\right)$$

where *σ*_Silica_ (= 1.1 × 10^−5^/°C) and *σ*_Coating_ (= −3.4 × 10^−4^/°C) are the thermo-optic coefficients of fused silica and the coating polymer, respectively, and *n*_Coating_ is the refractive index of the coating polymer around the microfiber. By substituting Equations (6) and (7) into Equation (5), the temperature sensitivity of the FSR in the polymer-overlaid microfiber MZI can be derived as:

(8) $$\frac{\partial (\Delta \lambda )}{\partial T}=-\frac{\lambda_{1}\lambda_{2}l_{1}}{{\left(\Delta \phi \right)}^{2}}\left(\underset{TE}{\underset{︸}{\alpha_{\mathrm{Coating}}n_{1}}}+\underset{TO}{\underset{︸}{\sigma_{\mathrm{Silica}}+\sigma_{\mathrm{Coating}}\frac{\partial n_{1}}{\partial n_{\mathrm{Coating}}}}}\right)$$

where the first and second terms in the parentheses correspond to the thermal expansion (TE) and thermo-optic (TO) terms, respectively, in the polymer-overlaid microfiber MZI. By using the finite element method (FEM), we analyzed the effect of the thermal expansion and the thermo-optic factors on the FSR variation in the proposed polymer-overlaid microfiber MZI as the external temperature increased. In Equation (8), it is clear that the thermal expansion factor (TE) can be readily achieved by considering *α*_Coating_ and the effective index (*n*_1_) of the polymer-overlaid microfiber. After theoretically evaluating the variation of the effective refractive index (*n*_1_) for the microfiber relative to variations in the refractive index of the low index polymer (*n*_Coating_), we obtained the value of ∂*n*_1_/∂*n*_Coating_ in order to analyze the total thermo-optic factor (TO) in Equation (8). Since the effective index of the microfiber with a strong evanescent field is highly sensitive to ambient index change, the value of ∂*n*_1_/∂*n*_Coating_ depends on the waist diameter of the microfiber [30]. 

**Figure 3:**
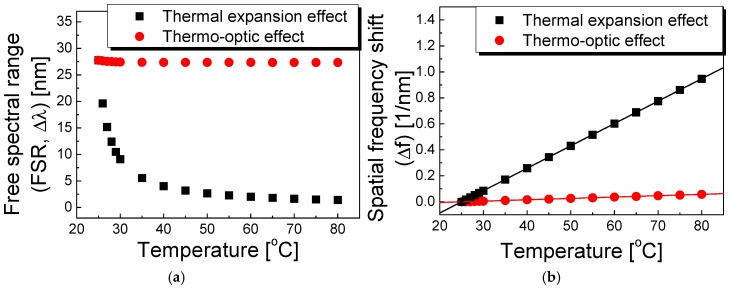
Theoretical results on the FSR variation (**a**) and the spatial frequency shift (Δ*f*) (**b**) as functions of the temperature by considering the thermal expansion and the thermo-optic effects.

Figure 3a presents the FSR variation in the polymer-overlaid microfiber MZI as a function of temperature when considering the thermal expansion and thermo-optic factors. It is evident that the temperature sensitivity of the FSR in the polymer-overlaid microfiber MZI is eventually negative, which is predominantly due to the thermal expansion factor (black square), as seen in Figure 3a. Increasing the external temperature consequently reduces the FSR of the polymer-overlaid MZI. Figure 3b shows the theoretical results of the spatial frequency shift as a function of temperature after applying FFT. Since the spatial frequency (Δ*f*) is inversely proportional to the FSR, the spatial frequency shifts to higher frequencies with increasing temperature. The temperature sensitivities of the spatial frequencies (Δ*f*) affected by the thermal expansion and the thermo-optic terms were theoretically estimated to be ~17.2 and ~1.05 pm^−1^ °C^−1^, respectively. It is notable that the contribution of the thermal expansion factor to the temperature sensitivity of the FSR in the polymer-overlaid microfiber MZI is ~16 times higher than that of the thermo-optic term.

## 4. Experimental Results and Discussion

**Figure 4:**
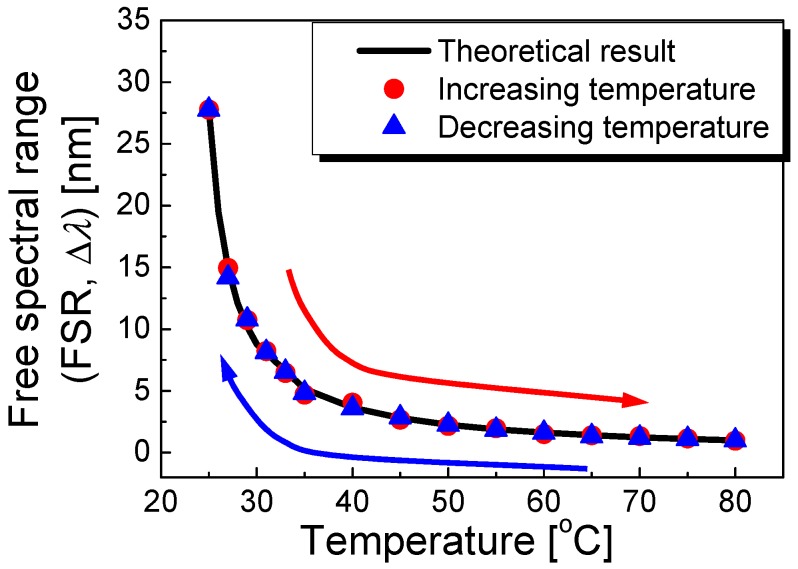
Theoretical (black line) and experimental results (red circles and blue triangles) on the FSR variation in the polymer-overlaid microfiber MZI as a function of temperature.

Figure 4 depicts the theoretical (black line) and experimental results (red circles and blue triangles) on the variation of the FSR in the proposed polymer-overlaid microfiber MZI as a function of temperature. The temperature dependence of the FSR shows a strong nonmonotonic behavior because the FSR of the polymer-overlaid microfiber MZI is inversely proportional to Δ*n*·*l*. As seen in Figure 4 (specifically the red circles), increasing the external temperature reduced the FSR of the polymer-overlaid microfiber MZI due to the enhanced thermal expansion and thermos-optic terms of the polymer-overlaid microfiber MZI. In contrast, decreasing the temperature increased the FSR of the polymer-overlaid microfiber MZI, as seen in Figure 4 (blue triangle). No differences in the FSR variations via increasing or decreasing the temperature were observed. The temperature sensitivity of the FSR was experimentally measured to be −8.29 nm/°C at a temperature of 25 °C, as shown in Figure 4. The theoretical results were in good agreement with the experimental ones.

**Figure 5:**
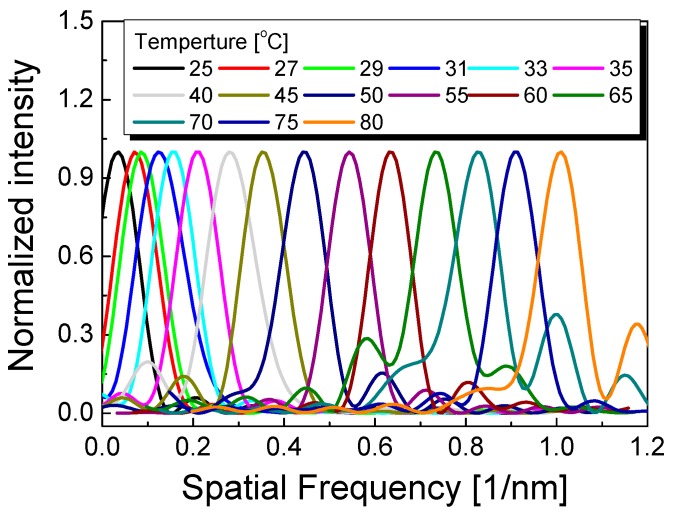
Spatial frequency spectra of the polymer-overlaid microfiber MZI with variations in temperature.

**Figure 6:**
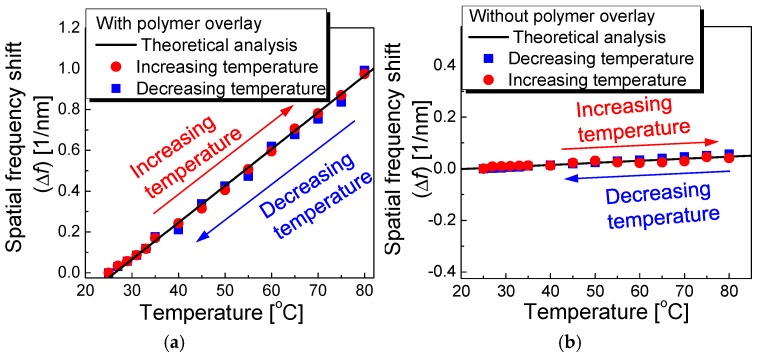
Spatial frequency shift (Δ*f*) (**a**) with and (**b**) without a polymer overlay as a function of temperature.

Figure 5 shows the spatial frequency spectra of the polymer-overlaid microfiber MZI (transformed using FFT) as the applied temperature increases. The zero padding method was applied to enhance the frequency resolution and analysis accuracy [31]. Figure 6a,b show the spatial frequency shift (Δ*f*) with and without polymer overlay, respectively, as a function of temperature. The nonmonotonic behavior of the temperature dependence of the FSR was effectively eliminated by considering the spatial frequency after applying the FFT, as seen in Figure 6a,b. As seen in Figure 6, increasing the temperature shifted the spatial frequency peak to higher frequencies (red circles) because of the decreased FSR in the polymer-overlaid microfiber MZI. Conversely, decreasing the temperature shifted the spatial frequency peak to lower frequencies (blue squares). However, no severe differences in the spatial frequency shifts when increasing or decreasing the temperature were observed. The temperature sensitivity of the spatial frequency peak of the polymer-overlaid microfiber MZI was higher than that without a polymer overlay. The temperature sensitivity of the spatial frequency in the polymer-overlaid microfiber MZI was measured to be 18.3 pm^−1^ °C^−1^, which was ~17 times higher than that of the microfiber MZI without polymer coating (1.04 pm^−1^ °C^−1^).

## 5. Conclusions

The temperature sensitivity of the FSR in a polymer-overlaid microfiber MZI was investigated by considering the thermal expansion and thermo-optic factors. After fabricating the microfiber with a waist diameter of ~10 μm, the microfiber was spin-coated with a low index polymer and eventually the polymer-overlaid microfiber MZI was configured. Since the microfiber has a strong evanescent field and a small waist diameter, the temperature sensitivity of the microfiber MZI was inevitably affected by the thermal properties of the low index polymer overlay. The effects of the thermal expansion and the thermo-optic factors on the temperature sensitivity of the FSR in the proposed polymer-overlaid microfiber MZI were theoretically analyzed. It was determined that the thermal expansion contribution to the temperature sensitivity of the FSR for the polymer-overlaid microfiber MZI was 16 times higher than that of the thermo-optic factor. The temperature sensitivity of the FSR in the polymer-overlaid microfiber MZI was successfully improved to −8.29 nm/°C at a temperature of 25 °C. After applying the FFT and zero padding methods, the optical spectra of the polymer-overlaid microfiber MZI was converted to spatial frequency spectra. The temperature sensitivity of the spatial frequency in the proposed polymer-overlaid MZI was found to be 18.31 pm^−1^ °C^−1^ in the temperature range of 25 to 80 °C, which was 17 times higher than that of the microfiber MZI without polymer coating (1.04 pm^−1^ °C^−1^).

## References

[1] Zhang Y.N., Peng H., Qian X., Zhang Y., An G., Zhao Y. (2017). Recent advancements in optical fiber hydrogen sensors. Sens. Actuators. B Chem..

[2] Zhang H., Wu Z., Shum P.P., Dinh X.Q., Low C.W., Xu Z., Wang R., Shao X., Fu S., Tong W. (2017). Highly sensitive strain sensor based on helical structure combined with Mach-Zehnder interferometer in multicore fiber. Sci. Rep..

[3] Liu Y., Wu G., Gao R., Qu S. (2017). High-quality Mach–Zehnder interferometer based on a microcavity in single-multi-single mode fiber structure for refractive index sensing. Appl. Opt..

[4] Huang X., Li X., Yang J., Tao C., Guo X., Bao H., Yin Y., Chen H., Zhu Y. (2017). An in-line Mach-Zehnder interferometer using thin-core fiber for ammonia gas sensing with high sensitivity. Sci. Rep..

[5] Xie N., Zhang H., Liu B., Song B., Wu J. (2017). Characterization of temperature-dependent refractive indices for nematic liquid crystal employing a microfiber-assisted Mach–Zehnder interferometer. IEEE Light. Technol..

[6] Li Y., Tong L. (2011). Mach–Zehnder interferometers assembled with optical microfibers or nanofibers. Opt. Lett..

[7] Li X., Ding H. (2014). Temperature insensitive magnetic field sensor based on Ferrofluid clad microfiber resonator. IEEE Photonics Technol. Lett..

[8] Lee Y., Moon J.S., Kim K., Oh J.W. (2017). Polarity Index Dependence of M13 Bacteriophage-Based Nanostructure for Structural Color-Based Sensing. Curr. Opt. Photonics.

[9] Wang Y., Yang M., Wang D.N., Liu S., Lu P. (2010). Fiber in-line Mach–Zehnder interferometer fabricated by femtosecond laser micromachining for refractive index measurement with high sensitivity. Opt. Soc. Am. B.

[10] Jiang L., Yang J., Wang S., Li B., Wang M. (2011). Fiber Mach–Zehnder interferometer based on microcavities for high-temperature sensing with high sensitivity. Opt. Lett..

[11] Wang X., Chen D., Li H., Feng G., Yang J. (2017). In-line Mach–Zehnder interferometric sensor based on a seven-core optical fiber. IEEE Sens. J..

[12] Duan V., Rao Y., Xu V., Zhu T., Wu D., Yao J. (2011). In-fiber Mach–Zehnder interferometer formed by large lateral offset fusion splicing for gases refractive index measurement with high sensitivity. Sens. Actuators B Chem..

[13] Gallego D., Lamela H. (2009). High-sensitivity ultrasound interferometric single-mode polymer optical fiber sensors for biomedical applications. Opt. Lett..

[14] Lim K.S., Harun S.W., Damanhuri S.S.A., Jasim A.A., Tio C.K., Ahmad H. (2011). Current sensor based on microfiber knot resonator. Sens. Actuators A Phys..

[15] Fang X., Liao C.R., Wang D.N. (2010). Femtosecond laser fabricated fiber Bragg grating in microfiber for refractive index sensing. Opt. Lett..

[16] Bai Z., Gao S., Deng M., Zhang Z., Li M., Zhang F., Liao C., Wang Y., Wang Y. (2017). Bidirectional bend sensor employing a microfiber-assisted U-shaped Fabry-Perot cavity. IEEE Photonics J..

[17] Yoon M.S., Kim H.J., Brambilla G., Han Y.G. (2012). Development of a small-size embedded optical microfiber coil resonator with high Q. J. Korean Phys. Soc..

[18] Wei F., Mallik A.K., Liu D., Wu Q., Peng G.D., Farrell G., Semenova Y. (2017). Magnetic field sensor based on a combination of a microfiber coupler covered with magnetic fluid and a Sagnac loop. Sci. Rep..

[19] Ding J., Zhang A.P., Shao L., Yan J., He S. (2005). Fiber-taper seeded long-period grating pair as a highly sensitive refractive-index sensor. IEEE Photonics Technol. Lett..

[20] Jasim A.A., Harun S.W., Arof H., Ahmad H. (2013). In line microfiber Mach–Zehnder interferometer for high temperature sensing. IEEE Sens..

[21] Liao C.R., Wang D.N., Wang Y. (2013). Microfiber in-line Mach–Zehnder interferometer for strain sensing. Opt. Lett..

[22] Tan Y., Sun L., Jin L., Li J., Guan B. (2013). Microfiber Mach-Zehnder interferometer based on long period grating for sensing applications. Opt. Express.

[23] Zhu S., Pang F., Wang T. Single-mode tapered optical fiber for temperature sensor based on multimode interference. Proceedings of the 2011 Asia Communications and Photonics Conference and Exhibition (ACP).

[24] Ahmed F., Ahsani V., Saad A., Jun M.B.G. (2016). Bragg Grating Embedded in Mach-Zehnder Interferometer for Refractive Index and Temperature Sensing. IEEE Photonics Technol. Lett..

[25] Yoon M.S., Park S., Han Y.G. (2012). Simultaneous measurement of strain and temperature by using a micro-tapered fiber grating. IEEE J. Light. Technol..

[26] Birks T.A., Li Y.W. (1992). The shape of fiber tapers. J. Light. Technol..

[27] Xu Y., Lu P., Chen L., Bao X. (2017). Recent developments in micro-structured fiber optic sensors. Fibers.

[28] Long W., Zou W., Hong Z., Su Y., Tong L., Yang L., Zhou L., Li X., Chen J. Characterization of DNA optical microfiber devices fabricated by drawing. Proceedings of the 2011 Conference on Lasers and Electro-Optics (CLEO).

[29] Lim T.Y., Kim Y.S., Park S.C. (2017). Achromatic and athermal design of an optical system with corrected Petzval curvature on a three-dimensional glass chart. Curr. Opt. Photonics.

[30] Wo J., Wang G., Cui Y., Sun Q., Liang R., Shum P.P., Liu D. (2012). Refractive index sensor using microfiber-based Mach–Zehnder interferometer. Opt. Lett..

[31] Chae Y.G., Park E.K., Jeon M.Y., Jeon B.H., Ahn Y.C. (2017). Stiffness comparison of tissue phantoms using optical coherence elastography without a load cell. Curr. Opt. Photonics.

